# Connecting communities to primary care: a qualitative study on the roles, motivations and lived experiences of community health workers in the Philippines

**DOI:** 10.1186/s12913-020-05699-0

**Published:** 2020-09-11

**Authors:** Eunice Mallari, Gideon Lasco, Don Jervis Sayman, Arianna Maever L. Amit, Dina Balabanova, Martin McKee, Jhaki Mendoza, Lia Palileo-Villanueva, Alicia Renedo, Maureen Seguin, Benjamin Palafox

**Affiliations:** 1 College of Medicine, University of the Philippines – Manila, Metro Manila, Philippines; 2 Department of Anthropology, University of the Philippines – Diliman, Quezon City, Philippines; 3 Faculty of Public Health & Policy, London School of Hygiene & Tropical Medicine, 15-17 Tavistock Place, London, WC1H 9SH UK

**Keywords:** Community health workers, Primary health care, Human resources for health, Health systems, Philippines

## Abstract

**Background:**

Community health workers (CHWs) are an important cadre of the primary health care (PHC) workforce in many low- and middle-income countries (LMICs). The Philippines was an early adopter of the CHW model for the delivery of PHC, launching the Barangay (village) Health Worker (BHW) programme in the early 1980s, yet little is known about the factors that motivate and sustain BHWs’ largely voluntary involvement. This study aims to address this gap by examining the lived experiences and roles of BHWs in urban and rural sites in the Philippines.

**Methods:**

This cross-sectional qualitative study draws on 23 semi-structured interviews held with BHWs from barangays in Valenzuela City (urban) and Quezon province (rural). A mixed inductive/ deductive approach was taken to generate themes, which were interpreted according to a theoretical framework of community mobilisation to understand how characteristics of the social context in which the BHW programme operates act as facilitators or barriers for community members to volunteer as BHWs.

**Results:**

Interviewees identified a range of motivating factors to seek and sustain their BHW roles, including a variety of financial and non-financial incentives, gaining technical knowledge and skill, improving the health and wellbeing of community members, and increasing one’s social position. Furthermore, ensuring BHWs have adequate support and resources (e.g. allowances, medicine stocks) to execute their duties, and can contribute to decisions on their role in delivering community health services could increase both community participation and the overall impact of the BHW programme.

**Conclusions:**

These findings underscore the importance of the symbolic, material and relational factors that influence community members to participate in CHW programmes. The lessons drawn could help to improve the impact and sustainability of similar programmes in other parts of the Philippines and that are currently being developed or strengthened in other LMICs.

## Background

Community health workers (CHWs) are an important cadre on the frontline of health systems in many low- and middle-income countries (LMICs). The 1979 Alma Ata Declaration on Primary Health Care (PHC), with its call for both more health workers and greater community participation [1], paved the way for CHWs to assume a greater range of functions, from health promotion to case management, with growing evidence of their increasing role which they have been shown to execute effectively and with good value for money [2].

**Fig. 1:**
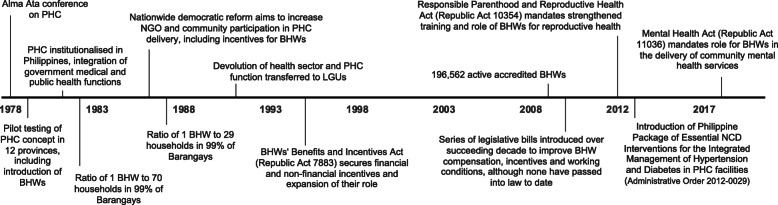
Timeline on the development of the Barangay Health Worker role in the Philippines (1978–2020). This figure illustrates the key events related to the introduction and developing role of Barangay Health Workers in the Philippines, since their introduction in the late 1970s to the present day. BHW Barangay Health Worker; CHW community health worker; LGU local government unit; NCD non-communicable disease; NGO non-governmental organisation; PHC primary health care

In many parts of the world, CHWs are seen as a means to deliver culturally appropriate health services to the community, serving as liaisons between community members and health care providers [3]. To achieve this, health systems and programmes typically enlist lay individuals with in-depth understanding of the culture and language of the communities from which they are drawn, with the expectation that they will require only minimal education and in-service training, although this will depend on their scope of work [4]. In 1981, the Philippines was one of the first countries to implement at scale the Alma Alta recommendation of PHC based on community participation (Fig. 1) [5].

Operating at the level of *barangays* or villages, the smallest unit of governance in the Philippines, volunteer Barangay Health Workers (BHWs) have evolved to become an essential component of the nation’s healthcare workforce [6–8] and have been key to the success of PHC in the country [5, 8]. In recognition of their contribution, the Philippine Congress passed the BHWs’ Benefits and Incentives Act (Republic Act 7883) in 1995 (Fig. 1), which is the most recent major reform to the BHW role. The law aimed to empower BHWs to self-organise, to strengthen and systematise their services to communities, and to create a forum for sharing experiences and recommending policies and guidelines [9]. The law also required local governments to offer benefits and allowances to BHWs, as well as scholarships for their children. The only constraint imposed by the law was that the number of BHWs could not exceed 1% of the community’s population. In practice, however, the number of BHWs, along with the scope of their responsibilities and the size of their allowances, are determined by the budget of the decentralised local government health board covering the barangay to which BHWs are assigned.

BHWs have now existed in the Philippines for almost four decades and have often been commended in evaluations of local health systems and community participation [6, 10, 11]. Yet, we lack a good understanding of what motivates and sustains their involvement on a largely voluntary basis. This understanding is crucial as the programme’s continued success and sustainability relies on its ability to motivate and mobilise community members to act as peer health advocates – and the difficulty of realising such community mobilisation has been noted [12]. The longevity of the Philippine BHW programme, especially when compared with more recent CHW models elsewhere, provides an excellent case study to explore these topics in depth.

This study aims to address this gap by documenting the experiences and roles of BHWs in selected urban and rural sites in the Philippines. We follow Campbell and Cornish’s approach that draws attention to relational and material aspects of the social context of participation, enhancing understanding of facilitators to community mobilisation to improve health [12]. This helps identify contextual dimensions often neglected in the literature that undermine or support community members’ motivation to participate in the BHW programme and sustain their involvement over time [12, 13]. As many countries are in the process of implementing new CHW programmes or strengthening existing ones, the findings from this study could inform ‘task shifting’ programmes and policies that seek to empower and mobilise communities to take more control over their health by means of CHWs [14], both in the Philippines and in other LMICs.

## Methods

This study was conducted as part of the Responsive and Equitable Health Systems-Partnership on Non-Communicable Disease (RESPOND) project, which uses longitudinal mixed-methods to better understand health system barriers to care for hypertension as a tracer condition for non-communicable diseases (NCD) in the Philippines [15]. The study was conducted in purposefully selected urban barangays in the City of Valenzuela and rural barangays in Quezon province, and data for this analysis was collected via semi-structured interviews with BHWs as part of the facilities assessment component of the RESPOND project.

### Data collection and management

A senior in-country, bilingual, social scientist researcher led the data collection and supervised two in-country, bilingual, trained research assistants (one male, one female) with relevant experience and backgrounds in communication and public health in administering semi-structured interviews in pairs in Filipino. A total of 23 BHWs were purposefully recruited, 13 from Valenzuela City and 10 from Quezon province, to maximize diversity of experience in terms of length of service, education and age, across the participating barangays. All BHWs in the study sites were women and those agreeing to participate in the study varied in age from 35 to 75 years. All but one were married. Their lengths of service ranged from 1 to 38 years, with 8 possessing 11 or more years of experience. Two participants reported recently returning to their duties following periods undertaking parental and household duties. The educational background of participants ranged from primary school to undergraduate degree. None received formal training as a health professional prior to starting their roles as BHWs.

The interview guide focused on their motivations for becoming a BHW, their day-to-day experiences of developing their role and responsibilities in the community, and their understanding of hypertension (Supplementary File 1). As BHWs in RESPOND project communities were engaged in the sampling of the household survey component, they were approached directly and oriented to the nature of the BHW study. Written informed consent was acquired from those who wished to participate, and interviews with each were arranged and conducted by the two research assistants in Filipino as the mutually shared language. Because all interviewees were women, it was considered important to include a female and male interviewer who could work flexibly to minimise response bias. Interviews were conducted and audio recorded in a secure place selected by participants between September 2018 and October 2019, lasting 30–60 min. After 15 interviews, data saturation was reached and subsequent interviews were conducted to ensure no new data was generated and to maximise sampling diversity.

Following each interview, written notes were reviewed jointly by the research assistants and BHWs to ensure accurate representation and interpretation. The two research assistants transcribed each interview recording verbatim in Filipino, and the fidelity transcriptions was assessed by the senior researcher against the recording. Anonymised transcripts were produced by removing all personal identifiers and attributes, and participants were assigned a pseudonym, which have been applied throughout this report. Research notes and signed consent forms were stored in locked cabinets accessible only to the research team. All digital audio recordings, digitised research notes, and original and anonymised transcript files were stored separately on secure, encrypted and password protected servers or laptops. All non-anonymised research material (e.g. audio recordings, original transcripts, notes) will be destroyed at project end, while consent forms and anonymised transcripts will be kept securely for 7 years thereafter.

### Data analysis and rigour

Verbatim transcriptions in Filipino were analysed using NVivo 12 software [16]. The senior social scientist led the open reading of the Filipino transcripts and several rounds of coding using a thematic approach [17] with the research assistants. The coding frame emerged, in part, inductively through multiple, iterative readings of the interview transcripts, but was also informed from our a priori interest in motivations and experiences of BHWs, drawing on Campbell and Cornish’s approach to examining how a “health enabling social environment” affects community mobilisation and participation [12]. After several rounds of coding, analytical memos of emerging and recurring themes were shared with the broader research team, who have expertise in primary health care, health system strengthening in LMICs and the local context, to conduct interpretation and contextualisation via regular discussions in English, ensuring the relevance and transferability of the results both locally and globally. This also included critical assessments of the findings’ plausibility, consistency with other research of findings, and in light of researchers’ own biases, preconceptions, preferences, and dynamic with the respondent (i.e. researchers were health professionals and/or staff of well-known universities) to ensure validity. Key themes, supporting quotations and statements included in memos (and subsequently in the manuscript) were extracted from interview transcripts and translated to English by the bilingual research assistants; and the quality of translations was assessed by bilingual senior researchers by checking and rechecking transcripts against the translated interpretations [18].

### Informed consent and ethical approval

Ethical approval for the research was obtained from the local research ethics board of the University of the Philippines Manila Panel 1. We obtained written informed consent from BHWs prior to the interview, ensuring that their anonymity, privacy and confidentiality would be maintained. BHWs were advised of their right to withdraw their participation at any time, although none of the participating BHWs did so.

## Results

In this section, we summarise the lived experiences of community members who volunteer as BHWs in our urban and rural study locations. We also describe the salient themes from these accounts that relate to factors that influenced their initial motivation to volunteer and that determine their continuing involvement.

### Becoming a BHW: the role of socio-political positioning and technical knowledge

The social relationships and political positioning of BHWs played an important role in their pathway to participation in the local health system (i.e. recruitment, appointment, and continuing inclusion). Recruitment was largely dependent on having these socio-political connections rather than on having the right skills or technical knowledge to deliver health services. The barangay captain, the leader of the village administration, holds the power to appoint BHWs, and with no formal guidelines to follow, appointments are arbitrary. Some BHWs recalled that they or their peers were appointed by the captain as a result of personal or political relationships, or following a recommendation from other barangay officials, including current BHWs or health staff. Some of the reasons cited for these endorsements included a history of active involvement in barangay activities, such as programmes on feeding, family planning, and fitness. For example, *Amy* (1 year in service) shared:

*I volunteered myself and I said to [the barangay councillor] that if he wins, [allot me a position]. I’ve been applying since before, but I was not given the opportunity. I only volunteer. When he won a seat, I finally got a position at the [health] centre. [The councillor] is my husband’s buddy*.

Importantly, however, there need not be any reason for the endorsement other than the prospective BHW’s need for a job, as *Ellen* (2 years in service) recalled:

*My livelihood then was to wash and iron clothes and take to care of children. But when I had a grandchild I could no longer do those tasks, so I asked the barangay treasurer (who happens to be my co-godmother) for any available jobs in the barangay. She told me that they can make me a BHW, so I suddenly became one.*

Ellen’s example points to the informality of the application process to become a BHW, something supported by most respondents’ accounts. *Cea* (11 years in service) recalled that she was interviewed by the local doctor and simply asked (not assessed) about her capacity to work in health centre: *“I was interviewed and she asked, ‘Can you do community area activities? Can you do duties in the health centre? Can you do all of this?’”* Skills and professional qualification, while useful, are largely secondary to personal connections.

Given that barangay captains are elected every 3 years and their power to appoint (or remove) BHWs, one’s position may not be secure when administrations change. Many BHWs recalled instances when they or their former peers were dismissed because they were not allied politically with the newly elected captain’s party. *Luisa* (5 years in service) shared that she was dismissed because her religious values did not permit her to vote; while *Catherine* (6 years in service) recalled that she was dismissed unexpectedly at an earlier point in her career:

*We thought that they would not remove anyone, including BHW positions. I was confident. I did not even vote and had no involvement in the political system. After the election on July 1, I went to the barangay office and my name was not included on the list of BHWs.*

While a connection to barangay officials appears to be a common route to becoming a BHW, involvement with the wrong politician or non-involvement in politics can also be liability, underscoring the political nature of the position. However, several examples of more merit-based appointments were noted, such as where applicants had previously volunteered for other community activities or programmes (e.g. in the barangay day care centre) or assisted existing BHWs.

### Mediating health: bridging and linking community members to services

In general, the activities performed by BHWs involved two roles: serving as frontline health centre staff and acting as community health mobilisers. However, the balance of activities depended on the priorities of the health centre manager to which the BHW was assigned. BHWs were commonly involved in various health centre programmes, including immunisation, maternal care, family planning and hypertension management. Their weekly schedules varied from barangay to barangay, but they typically spent the whole day in health centres 2–3 times a week.

As frontline staff at local health centres, BHWs are often the first point of contact for patients. They welcome patients and perform a range of specific tasks, including admitting and interviewing patients and recording patient information and/or vital signs (e.g. blood pressure), before being seen by a doctor or nurse, if available. BHWs confirmed that their role did not involve diagnosing or prescribing.

As community health mobilisers, BHWs serve as a bridge between the community and their local health centre, promoting health and engagement with existing services, often working house-to-house. They particularly encourage uptake of programmes such as child feeding and NCD prevention and screening at health centres. While they are not allowed to dispense medicines, administer vaccines, or provide direct patient care, they play a supportive role, which includes assisting midwives, blood pressure monitoring, and talking to and motivating patients to adopt appropriate health behaviours. *Gina (*38 years in service) shared:

*We encourage them. This is our job: to encourage them that we have a health centre and to seek help if they feel something.*

BHWs also assist patients in the community with self-management of their chronic conditions. For instance, they measure the blood pressure of those with hypertension at both the health centre and during house-to-house visits, take the opportunity to remind patients of upcoming follow-up appointments, advise them if medicines are available at the health centre for prescription refills, and educate community members. *Ruby* (22 years in service) shared:

*I remind them that they should not be confident if they don’t feel anything [symptoms]. We don’t know if we have hypertension.*

BHWs’ role as community health mobilisers also includes a public health surveillance component, following up on non-adherence and surveying prevailing health conditions in the community. *April* (8 years in service) described:

*If we are not in the health centre, we visit our assigned area. We ask who is pregnant. We ask who is sick. We ask who has tuberculosis. We also do lectures on tuberculosis.*

*Denden* (10 years in service) also described:

*We visit them. We knock on their doors and ask why they don’t visit the centre. We remind them to finish the programme. If they give us a chance, we explain the need to continue the programme. It’s like the patient and I are a tandem.*

BHWs’ local knowledge and position in the community are useful assets in their role as health mediators, helping them to identify health needs and engage with community members to link them to services*. Maria* (2 years in service) talked about using her local knowledge and position in the community to achieve this:

*We know for example in our community who has tuberculosis. We always research them, so that we encourage them to undergo treatment. During immunisation, we notify parents to bring their child to the health centre.*

BHWs also mentioned that they are often approached by patients before they have reached the health centre, which suggests that they enjoy a high level of trust among community members as intermediaries of the health system. *Lili* (11 years in service) told us about being contacted often by patients asking for medicines and using this opportunity to remind then about the importance of engaging with services to “*consult the doctor before taking medicine. It’s just not about taking medicine.”*

### Contracting arrangements and compensation

BHWs are considered part-time, volunteer workers and not government employees. Hence, they do not receive a regular salary. However, BHWs from rural areas reported being given honoraria and allowances of PhP 1150 (USD24) each month; in urban communities honoraria were also paid but their size, and that of any other allowances, varied depending on whether they were contracted by city or barangay administrations, with the latter having smaller budgets. Although urban BHWs all perform similar duties and report to local health centres, the financial incentives, in the form of honoraria to acknowledge their voluntary contributions and allowances to cover the incidental costs of carrying out their assignments (e.g. transport), varied by location. For barangay-funded BHWs, the combined lump sum was reported as PhP 2300 (USD 50) per month distributed in cash by barangay offices, and PhP 3000 (USD 60) for city-funded BHWs paid through a designated local bank. In addition to honoraria and allowances, city-funded BHWs are provided with PhilHealth membership, the national social health insurance programme.

Other non-monetary incentives that BHWs reported receiving included free medicines from the health centre, free health services, and groceries at Christmas from local or barangay administrations. Since the honoraria received by both rural and urban BHWs is insufficient to support themselves and their families, most respondents reported also having part-time jobs, mostly in the service industry, alongside their BHW duties.

### Beyond economic empowerment: social positioning and common good

We now describe how relational dimensions of BHWs’ work play an important role in their initial motivations and in sustaining participation over time. Interviewees described a range of motivations for volunteering as BHWs, with the desire to serve the community and improve its health as the most frequently mentioned factor. *Gina* (38 years in service) described this motivation to contribute to the common good of the community:

*I observed the lack of health [knowledge] in our barangay. Parents are not aware of what to do for their child’s fever. They only cover them with [wet towels]. It's just like a cold. I want to know why, why they lack attention and knowledge.*

*Sisa* (1 year in service) cited similar motivation and particularly wanted to improve health-seeking behaviour of the community: *“I want the community to be aware that if they are sick, they should consult a doctor. I advise them to go to the doctor.” Jhoanne* (4 years in service) derived pleasure from serving the community: *“I’m happy to serve my fellow community members. You will be happy if you do it with you heart. You will learn a lot [from being a BHW].”*

Supporting the community required some BHWs to contribute their own money, for example to purchase medicines for patients who could not afford them, and to cover costs to travel to their assigned areas. *April* (8 years in service) described the honorarium and allowances provided as insufficient to shoulder such expenses:

*During our areas of assignment, it’s our own-pocket expenses. It’s fortunate if the barangay can provide a transportation service. What if none? We will walk and of course, we will eat and drink. Not all households can provide drinks. Our PhP 3000 honorarium [and allowance] is really not enough.*

*Gina* (38 years in service), said that it was inevitable that she would use her own funds:

*I visited a patient and he had no food. I gave my own money. I also arrived when he was sick. He had no money for medicine and I gave him money. I accompanied a patient to the hospital. It’s my own pocket expense.*

*Mell* (5 years in service) described how a provincial governor promised to increase the financial incentives given to BHWs.

*Our governor’s term is about to end, but he promised that we, the BHWs, will become counterparts of nurses, doctors and midwives. We need salary. We need honorarium.*

Although some BHWs reported struggling financially as a consequence of the low honorarium and allowances, they still expressed contentment with what they were doing. The opportunity to serve the community gave them a sense of fulfilment, through the relational aspects of their involvement in the programme. Their relationships with other BHWs, patients, and the wider community, as well as the new knowledge they gained, compensated for the relative lack of financial and non-financial incentives. *Denden* (10 years in service) expressed that it was not about how high her compensation was:

*If feels good to help. Sometimes [patients] comfortably share their stories. That’s the best part. After they are treated, they go again to you and say thank you. That’s the best part to us. A simple thank you means a lot and it makes us smile. It’s not about how high is our compensation. If you enjoy your work, it’s the best feeling. It’s feels good to give service to the community.*

Enhancing one’s social position, particularly through establishing new relationships in the community, gaining respect, and acquiring technical knowledge, played an important role in sustaining participation. *Amy* (1 year in service) echoed: *“Patients trust us. One of my neighbours visited my house and asked if I can take her blood pressure or when I will next be on duty. [I feel] they trust me. They wait for me to be on duty.”*

Cherry (12 years in service) shared that she gained respect (‘*respeto*’) from being a BHW:

*Interviewer: What do you feel being a BHW? Are you happy?*

*Cherry: “I’m happy that they address me as ‘Ma’am’. If I was not a BHW, they would not address me as ‘Ma’am’. I’m happy with that. They respect me. I gain respect.”*

Many BHWs spoke of the opportunities to travel outside of their localities, develop camaraderie with fellow BHWs, and acquire health knowledge as rewards in themselves, pointing to the role conferring a multiplicity of benefits. As *Lili* (11 years in service) said:

*Being a BHW is difficult, but fun, because you are able to visit places you don't get to visit for seminars, out of town activities, and the like. And then of course the ‘bonding’ here in the health centre. It’s also fun because we learn a lot.*

This camaraderie also appeared to be developed and reinforced through the model of BHW training, which was similar in both urban and rural study locations. New recruits typically shadowed more experienced BHWs and other health workers to familiarise themselves with health centre workflows. This was followed by brief training on basic procedures, such as blood pressure monitoring and first-aid. BHWs gained further knowledge and skills through participating in occasional activities organised by national and/or local government agencies, including workshops on immunisation, tuberculosis management and monitoring, and basic life support, among others. While BHWs found such activities useful, many claimed that the most valuable sources of knowledge and skills came from their interactions with experienced BHWs and from their own experiences on the job.

Finally, since the BHWs interviewed were typically mothers and wives, they also found the additional income and, as mentioned above, the opportunity to gain health knowledge and skills as attractive incentives. As *Sisa* (1 year in service) recalled:

*I’m a mother and for my children, it’s good that I have [health] knowledge. I have no husband and I mainly guide my children. I need [health] knowledge in case of emergency. I can use what [I learn] as a BHW and apply it to my family.*

## Discussion

This paper examines the experiences of local women in urban and rural locations of the Philippines involved in the delivery of primary care as part of the national BHW programme, a four-decade-long experiment in community participation. By focussing on the socio-political and material conditions that facilitate and sustain their involvement in the programme, as advocated by Campbell and Cornish [12], the findings from this case study identify factors that contribute to the continued success and longevity the BHW programme in these settings. Such findings may improve the impact and sustainability of similar programmes in other parts of the Philippines and other LMICs. Below, we use the concepts suggested by Campbell and Cornish to contextualise our results [12].

### Symbolic context

Regarding the symbolic context, which refers to relevant meanings, ideologies or worldviews that shape community perceptions of the BHW programme, the participants’ accounts indicate that the BHW role is respected by community members and confers social status, which are two widely recognised factors known to motivate individual CHWs [19]. Those interviewed in both rural and urban locations noted that community members valued them as resource persons for health, and as peer supporters who assisted others to navigate the health system and manage their health conditions. These symbolic meanings attached to the BHW role are also formally acknowledged and reinforced in several ways. First, the BHW role is defined in national law, which recognises them as essential components of the national health workforce with specific rights and responsibilities [9]. Also, the value of BHW contributions to primary care service delivery is embodied in the monetary compensation (i.e. honorarium) mandated by the law and the various non-monetary incentives provided to them. That many of the interviewees became BHWs through appointment by community officials further signals the perceived status attached to the role.

While the respect conferred by each of the symbolic factors noted above motivated many participants to initially seek and maintain their BHW appointment, the same factors were also found to have certain stigmas attached, which could discourage community members from becoming BHWs. The commonly held view that BHW appointments are politicised or require personal connections to local officials poses a barrier to wider community participation, leading to an inequitable distribution throughout the community of the health and social benefits derived from the BHW programme. The resulting turnover of BHW staff at each electoral cycle also negatively affects the sustainability and effectiveness of the programme, as resources invested into training BHWs and building rapport within the community are lost with each new round of appointments. This also negatively impacts the ‘embeddedness’ of BHWs in the community and their integration into local health systems, which are recognised enablers to CHW programme success [2]. It is notable that reforming the BHW appointment process was recommended as far back as the early 1990s [20]. Furthermore, the national BHW law codifies the role as ‘voluntary’, despite the recognition of the essential contributions that they make to the health system [9]. While not explicitly mentioned by any participants during interviews, some may question why such an essential role is only voluntary, rather than salaried.

Our observation that the BHWs engaged in all of our study sites were exclusively female points to yet another symbolic factor that may limit wider participation and the impact of the programme: the persistent effect of cultural patriarchy on women’s labour force participation in the Philippines. Despite the country’s world-leading performance on several key indicators of gender equality, the most recent figures for 2019 indicate that just under half of all Filipinas above 15 years of age are economically active, placing them in bottom third of over 180 nations [21]. Moreover, these women’s jobs are largely restricted to those considered as extensions of the mothering, caring and educating roles defined by a patriarchal worldview [22, 23]. The descriptions of the BHW role and factors motivating women to seek BHW appointments are consistent with this worldview, which likely explains the absence of male participation and the role’s categorisation as voluntary, as has been observed in numerous CHW programmes in both lower and higher income country settings [24]. While BHWs felt respected by community members, those who adhere to patriarchal views may not consider BHWs as sufficiently authoritative to trust or follow any health advice given, further eroding BHW’s embeddedness in the community and their impact of community health [2].

### Material context

Participants in both rural and urban communities unanimously valued the various resources they were able to access as BHWs. These resources comprise Campbell and Cornish’s material context, which empowers community members to put themselves forward for appointment as BHWs [12]. Several described how the health knowledge and skills acquired as BHWs not only allowed them to perform their assigned tasks effectively, but also enhanced their roles as the carers and educators of family and friends. And while many protested the paltry level of monthly honorarium and allowances given to BHWs, this financial benefit was still considered a useful source of primary or secondary income; however, we acknowledge that this may be due to the fact that our participants were assigned to and drawn from low-income communities. These findings align closely with existing evidence, which also demonstrates clear positive links between incentive levels (both monetary and non-monetary) and CHW motivation, performance and retention [2, 19, 25].

The decentralisation of decision-making powers for the delivery of health care from national down to provincial, city/municipal and even barangay administrative levels [26] also appears to influence the material context of the BHW’s daily working conditions. This is most evident in the incentive packages that varied depending on the governance level to which the BHW was attached. Such decentralisation means that the amounts of local government budgets allocated to health, and primary care specifically, depends largely on the priorities of locally elected officials, which likely varies from jurisdiction to jurisdiction and administration to administration. This, in turn, is known to directly affect CHW’s scopes of work, remuneration and incentive levels, training and supervision, and logistical and material support (e.g. transport, medicines, equipment, etc.) needed for them to perform their duties – all of which impact their motivation, performance and retention, ultimately determining the effectiveness of CHW programmes [2, 7, 20, 27, 28]. Our findings suggest that BHW monetary incentives should be reviewed periodically by decentralised decision-makers to ensure that their levels are appropriate for their specific contexts and scopes of work, as has been advocated by several studies [29, 30]. Also, ensuring health centres are continuously stocked with medicines and supplies will support BHW activities and foster the trust and confidence that community members have both in BHWs and in local health services.

Finally, while it is acknowledged that CHWs in LMICs can effectively support a range community-based programmes targeting NCDs, including tobacco cessation, diabetes and hypertension control [31], evidence emerging from mainly high-income settings also suggests that, with sufficient training, supervision and definition in roles, they may also be effectively integrated into the provision of other primary care services, including mental health and drug rehabilitation [2, 32]. These issues have been prioritised by national government as reflected in several key reforms since 2012 that have mandated the involvement of BHWs in community services for mental health, hypertension, diabetes and addiction (Fig. 1) [33–35]. However, CHWs should not be used as a remedy for reducing the burden of other health workers or other symptoms of a weak health system [36]. Also, when broadening CHW responsibilities, careful consideration must be given to the education, training, remuneration and commitment required from CHWs to deliver such services, as such parameters vary from programme to programme, even within countries as described above. Importantly, programmes must ensure that such expansion does not result in task overload, which could reduce productivity and worsen health population health outcomes [37].

### Relational context

Perhaps the factors that have contributed most to the success and longevity of the BHW programme in the Philippines pertain to the Campbell and Cornish’s relational context, which are the features that encourage community participation through the prospect of being involved in leadership, decision-making, and the building of social capital [12]. As above, the respect from community members that the BHW role confers is derived not only from the symbolic, but also from other features that mark out these individuals as community leaders. In our study communities, BHWs viewed themselves as ‘local’ health experts, peer mentors and trainers, and brokers and facilitators of patient care and access to the local health system, particularly for the underserved and marginalised in their communities, all of which are well documented nonmonetary CHW incentives [19]. These functions appeared to underlay the profound satisfaction they derived from their position, despite the perceived inadequacy of material remuneration. It is also evident that these leadership functions succeed by fostering the development of social capital in both its bonding form (by helping community members to “get by” and benefit from existing health services), and its bridging form (by helping other BHWs to “get ahead” and succeed in the role) [38].

Recent research has, indeed, clarified the significance of social capital for the CHW role. One review concludes that the CHW’s ability to affect positive health behaviour change rests largely on the bonding and bridging social capital existing between them and community members [39]. Others have discussed how the social capital wielded by CHWs in these forms is crucial to facilitating access to care in poor and marginalised communities [40]. Again, these notions resonate clearly with the experiences and motivations mentioned by respondents in both rural and urban study locations. With continuing urban migration, the rising burden of NCDs, and the immense strain these trends are placing on the health system both in the Philippines and beyond, the value CHWs and the social capital that they bring is only likely to grow in importance [41].

However, our findings suggest that more attention could be given to BHW involvement in decision-making about their role and primary care more generally, which itself constitutes a form of linking social capital as a means of spanning power divisions between community members and those who design and fund community health services [38]. Despite being explicitly mandated by Republic Act 7883 [9], the participant accounts from our study locations provided little evidence that such involvement occurred in any institutionalised form. Meaningful participation of BHWs in decision making represents yet another means of integrating and embedding them further into the local health system [2, 40]. In the decentralised Philippine context, this could be readily achieved, for example, through the inclusion of BHWs as ‘local’ health experts in multi-stakeholder consultations administered by local governments on the planning, financing, implementation, management and monitoring of community health services [42]. With the ongoing implementation of the Universal Health Care Act in the Philippines [43], and the renewed commitment to strengthen primary health care [44], a formidable cadre of BHWs stand ready to dedicate their time, energy and expertise to help realise these goals for the nation.

## Conclusion

The Philippine experience of integrating CHWs in the delivery of effective PHC over nearly four decades provides an important, yet under-reported, case study of community participation and people-centred care. As many countries work to develop and strengthen CHW programmes in their effort to achieve universal health care and the health-related sustainable development goals, the lessons drawn from the Philippines could help to ensure that such programmes achieve optimal impact and sustainability.

## Supplementary information

**Additional file 1.** Topic guide for BHWs.

## Data Availability

The data that support the findings of this study are available on request from the corresponding author. The data are not publicly available due to the presence of information that could compromise research participant privacy and confidentiality.

## Abbreviations

BHW: Barangay (village) Health Worker
CHW: community health worker
LGU: local government unit
LMIC: low- and middle-income country
NCD: non-communicable disease
NGO: non-governmental organisation
PHC: primary health care
PhP: Philippine Peso
RESPOND: Responsive and Equitable Health Systems – Partnership for NCDs
USD: United States Dollar
