# Supplementary material for: Connecting communities to primary care: a qualitative study on the roles, motivations and lived experiences of community health workers in the Philippines
Source: BMC Health Serv Res. 2020 Sep 11;20:860. doi: 10.1186/s12913-020-05699-0 (PMC7488850; doi:10.1186/s12913-020-05699-0)
Supplement: Supplementary file 1 — Additional file 1. Topic guide for BHWs. [file 12913_2020_5699_MOESM1_ESM.docx]

**SUPPLEMENTARY MATERIAL: TOPIC GUIDE FOR BHWs**

**PART 1: Life of BHW**

1. What are the everyday activities of a BHW?
2. Usually, who are the BHWs? How many of you are there?
3. What are the benefits? Compensation?
4. What are the biggest challenges? (Material, financial, social)
5. How to become a BHW? How long have you been a BHW?
6. What influenced your initial motivation to volunteer and continuing involvement as a BHW?

**PART 2: Noncommunicable diseases**

1. What is your understanding of NCDs? HPN?
2. How do you deal with community members with HPN?
3. Are there existing programs to address NCDs and HPN?
4. To whom do you refer the patients? Where do patients go?
5. What do you think are the reasons why patients have difficulty with their NCDs, particularly HPN?

**PART 3: The health landscape**

1. What are the common health problems?
2. What do people do when they have an illness? (e.g. if illness is mild and severe)
3. Are there traditional medical practitioners here? What about alternative medicine?
4. What are beliefs about hypertension in particular, and sickness in general?
5. Do people use herbs? Supplements?
